# Resolution of subdural hemorrhage following interventional treatment of superior vena cava occlusion in a hemodialysis patient: a case report

**DOI:** 10.3389/fcvm.2025.1540854

**Published:** 2025-06-13

**Authors:** Qiquan Lai, Ling Chen, Xuejing Gao, Xi Zhang, Ziming Wan

**Affiliations:** Department of Nephrology, Urological Metabolism and Immunoecology Laboratory, The First Affiliated Hospital of Chongqing Medical University, Chongqing, China

**Keywords:** superior vena cava syndrome, subdural hemorrhage, end-stage renal disease, dialysis, case report

## Abstract

Superior vena cava syndrome (SVCS) and nontraumatic subdural hemorrhage (SDH) were rare but severe complications in hemodialysis patients. This case report presented a 73-year-old male with end-stage renal disease (ESRD) on long-term hemodialysis who developed SVCS due to central venous occlusion, subsequently complicated by SDH. After interventional treatment of SVCS with balloon angioplasty and stent placement in the left brachiocephalic vein and SVC, both the SVCS and SDH were resolved. This case gave hint to the pathophysiological connection between venous congestion and SDH, as the obstruction of the SVC led to increased intracranial venous pressure, contributing to SDH. Additionally, anticoagulation and dialysis-induced hemodynamic fluctuations may exacerbate the condition. This report highlights the need for timely intervention to relieve venous congestion in SVCS, which not only restores vascular access but also prevents severe neurological complications like SDH in hemodialysis patients.

## Introduction

Hemodialysis was a life-saving treatment for patients with end-stage renal disease (ESRD), yet it was associated with a range of serious complications. Among these, superior vena cava syndrome (SVCS) and nontraumatic subdural hemorrhage (SDH) were rare but life-threatening conditions that could significantly worsen clinical outcomes (1). SVCS typically resulted from central venous catheter-related stenosis or thrombosis, leading to venous hypertension and upper body congestion (2, 3). Meanwhile, SDH in dialysis patients was often attributed to uremia-induced platelet dysfunction, anticoagulation therapy, and hemodynamic instability during sessions (4, 5). Notably, long-term hemodialysis may cause cerebral atrophy and cognitive impairment, potentially mimicking or obscuring early symptoms of SDH and delaying diagnosis (6).

Although both conditions had been individually described, their simultaneous occurrence in a single patient had not been previously reported. The case presented here was unique in that the hematoma resolved following interventional treatment of the venous obstruction, which suggested a possible pathophysiological link between elevated intracranial venous pressure due to SVCS and the development of SDH (7). The case further underscored the importance of recognizing and promptly treating SVCS in dialysis patients, not only to restore vascular access but also to potentially prevent severe neurological complications.

## Case presentation

This case report was prepared in accordance with the CARE guidelines to ensure transparency and completeness in reporting. Written informed consent was obtained from the patient for the publication of any potentially identifiable images or data included in this article.

A 73-year-old male patient was diagnosed with ESRD secondary to chronic glomerulonephritis in 2014, initially presented with nausea and fatigue. Laboratory tests revealed a serum creatinine level of 728 μmol/L. Regular hemodialysis was initiated via a right internal jugular vein catheter as the primary vascular access. This patient had no history of primary hypertension, diabetes mellitus, or stroke. During long-term dialysis, the patient experienced episodes of renal hypertension and took antihypertensive medications intermittently, including sacubitril/valsartan and extended-release nifedipine. Blood pressure was generally maintained below 160/100 mmHg. In 2019, due to persistent dysfunction, the catheter was removed, and a left brachiocephalic arteriovenous fistula (AVF) was created in the left elbow, providing symptomatic relief and restoring adequate vascular access for hemodialysis. During each dialysis session, low-molecular-weight heparin (nadroparin 4,100 IU) was used for anticoagulation. Human erythropoietin was administered continuously and adjusted based on laboratory results to correct dialysis-related renal anemia. In addition, medications for managing calcium-phosphorus metabolism disorders were also prescribed if needed.

By March, 2021, this patient was admitted our institution because of mild swelling of the face, neck, and upper extremities. This patient complained of fatigue, poor appetite, mild headache but exhibited no motor deficits or altered consciousness. From the creation of the left brachiocephalic AVF in 2019 until the onset of symptoms, the patient underwent hemodialysis three times per week, with each session lasting four hours. Dialysis adequacy was monitored quarterly using Kt/V, which consistently ranged between 1.2 and 1.4, indicating adequate dialysis dose during this period.

**Figure 1:**
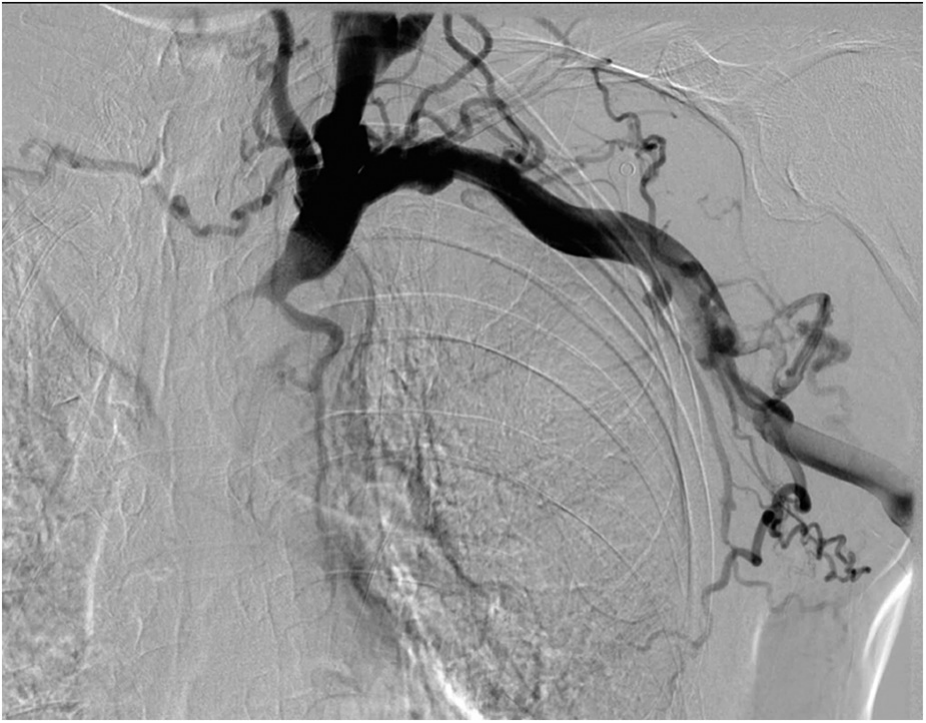
Digital subtraction angiography image showing occlusion of the left brachiocephalic vein and part of the superior vena cava.

**Figure 2:**
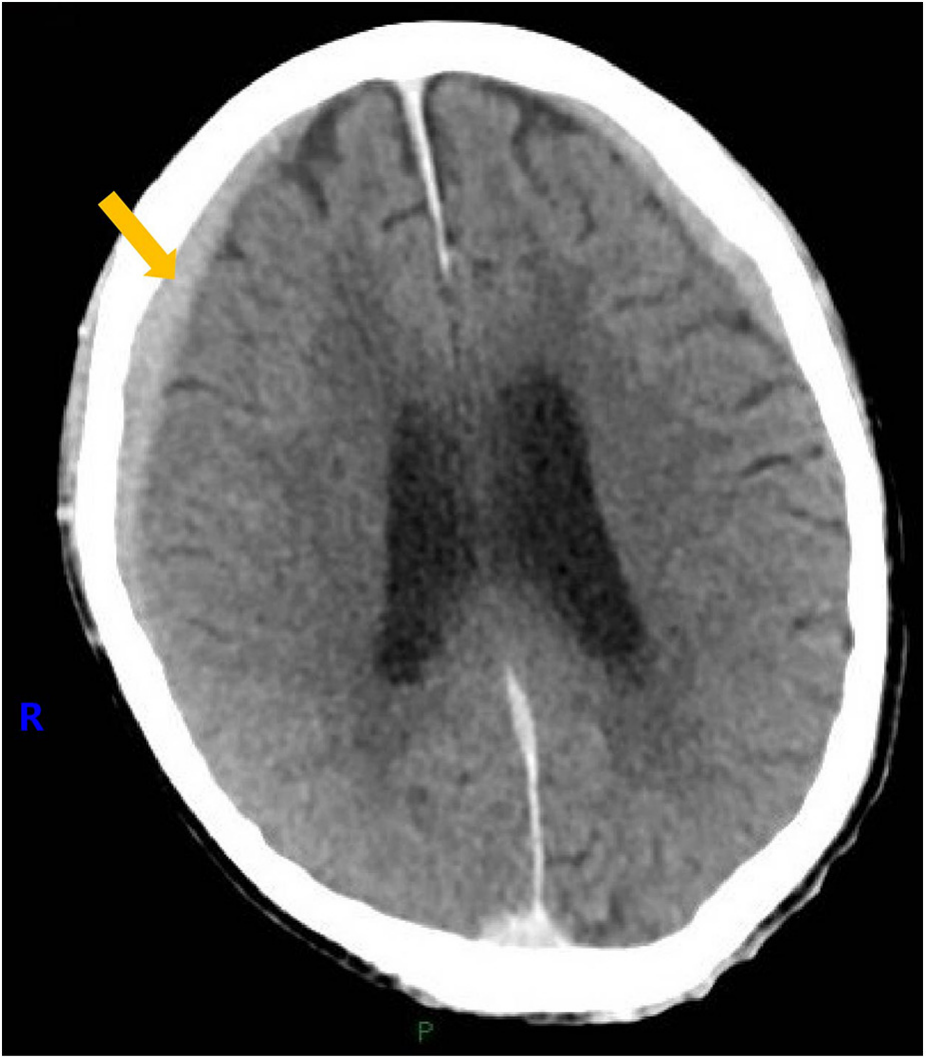
Cranial computed tomography image of a thin subdural hematoma in the right frontal and temporal region.

Upon admission, digital subtraction angiography revealed central venous occlusion involving the left brachiocephalic vein, and part of superior vena cava (SVC), accompanied by engorgement of the azygos vein, confirming the diagnosis of SVCS (Figure 1). Further cranial CT revealed a thin SDH in the right frontal and temporal region (Figure 2). Key laboratory evaluations were performed to assess hematologic and coagulation status (Table 1). The complete blood count revealed a white blood cell count of 2.55 × 10⁹/L, hemoglobin of 131.0 g/L, hematocrit of 40.8%, and platelet count of 130 × 10⁹/L. Coagulation profile showed a prothrombin time of 12.2 s, international normalized ratio of 1.03, activated partial thromboplastin time of 27.8 s, and thrombin time of 17.7 s, all within normal ranges. Fibrinogen was measured at 2.42 g/L, and D-dimer was 0.53 mg/L FEU, indicating no evidence of active coagulopathy or fibrinolysis. These findings suggested a relatively stable coagulation profile despite the presence of SDH.

**Table 1:** Summary of the patient's laboratory tests on admission.

Complete blood count		Coagulation profile	
White blood cell count	2.55 × 10⁹/L	Fibrinogen	2.42 g/L
Red blood cell count	3.61 × 10^12^/L	Fibrin degradation products	1.4 μg/ml
Hemoglobin	131.0 g/L	D-dimer	0.53 mg/L FEU
Hematocrit	40.8%	Liver and renal function tests	
Mean corpuscular volume	113.0 fL	Total protein	65 g/L
Mean corpuscular hemoglobin	36.3 pg	Albumin	42 g/L
Mean corpuscular hemoglobin concentration	321.0 g/L	Total bilirubin	13.1 μmol/L
Platelet count	130 × 10⁹/L	Direct bilirubin	12.5 μmol/L
Platelet distribution width	11.3 fL	Indirect bilirubin	0.6 μmol/L
Mean platelet volume	10.4 fL	Alanine aminotransferase	13 U/L
Plateletcrit	0.14%	Aspartate aminotransferase	18 U/L
Neutrophil percentage	56.8%	Alkaline phosphatase	61 U/L
Lymphocyte percentage	25.9%	Gamma-glutamyl transferase	26 U/L
Monocyte percentage	13.3%	Lactate dehydrogenase	374 U/L
Eosinophil percentage	2.4%	Cholinesterase	5,474 U/L
Basophil percentage	1.6%	Prothrombin time ratio	10.6 mmol/L
Neutrophil count	1.45 × 10⁹/L	Creatinine	659 μmol/L
Lymphocyte count	0.66 × 10⁹/L	Uric acid	403 μmol/L
Monocyte count	0.34 × 10⁹/L	Calcium	2.35 mmol/L
Eosinophil count	0.06 × 10⁹/L	Magnesium	0.92 mmol/L
Basophil count	0.04 × 10⁹/L	Phosphate	1.20 mmol/L
Coagulation profile		Lipid profile	
Red cell distribution width	14.9%	Total cholesterol	2.61 mmol/L
platelet large cell ratio	27.60%	Triglycerides	0.85 mmol/L
Prothrombin time	12.2 s	High-density lipoprotein cholesterol	0.88 mmol/L
Prothrombin time ratio	1.03	Low-density lipoprotein cholesterol	1.38 mmol/L
International normalized ratio	1.03	Apolipoprotein A1	1.05 g/L
Prothrombin activity	93.3%	Apolipoprotein B	0.49 g/L
Activated partial thromboplastin time	27.8 s	Lipoprotein *α*	279 mg/L
Thrombin time	17.7 s	High-sensitivity C-reactive protein	2.79 mg/L

**Figure 3:**
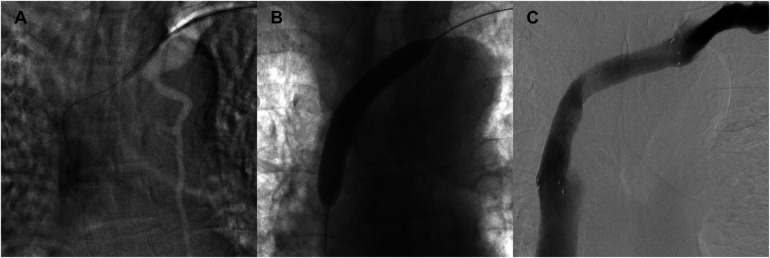
Revascularization of the left brachiocephalic vein and superior vena cava through balloon angioplasty and stent placement. **(A)** Initial image showing a guidewire successfully crossing the occluded segment of the left brachiocephalic vein. **(B)** Balloon angioplasty performed to dilate the occluded segment of the vein. **(C)** Post-stent image showing restored venous patency and contrast flow through the left brachiocephalic vein and superior vena cava.

To address SVCS, the patient underwent central venography, followed by balloon angioplasty and stent placement for revascularization of the left brachiocephalic vein and SVC on March 23, 2021 (Figure 3). Two days after revascularization, considering the limited cannulation space of the original AVF, a prosthetic arteriovenous graft was subsequently placed at the left elbow to establish more reliable vascular access for ongoing dialysis treatment.

**Figure 4:**
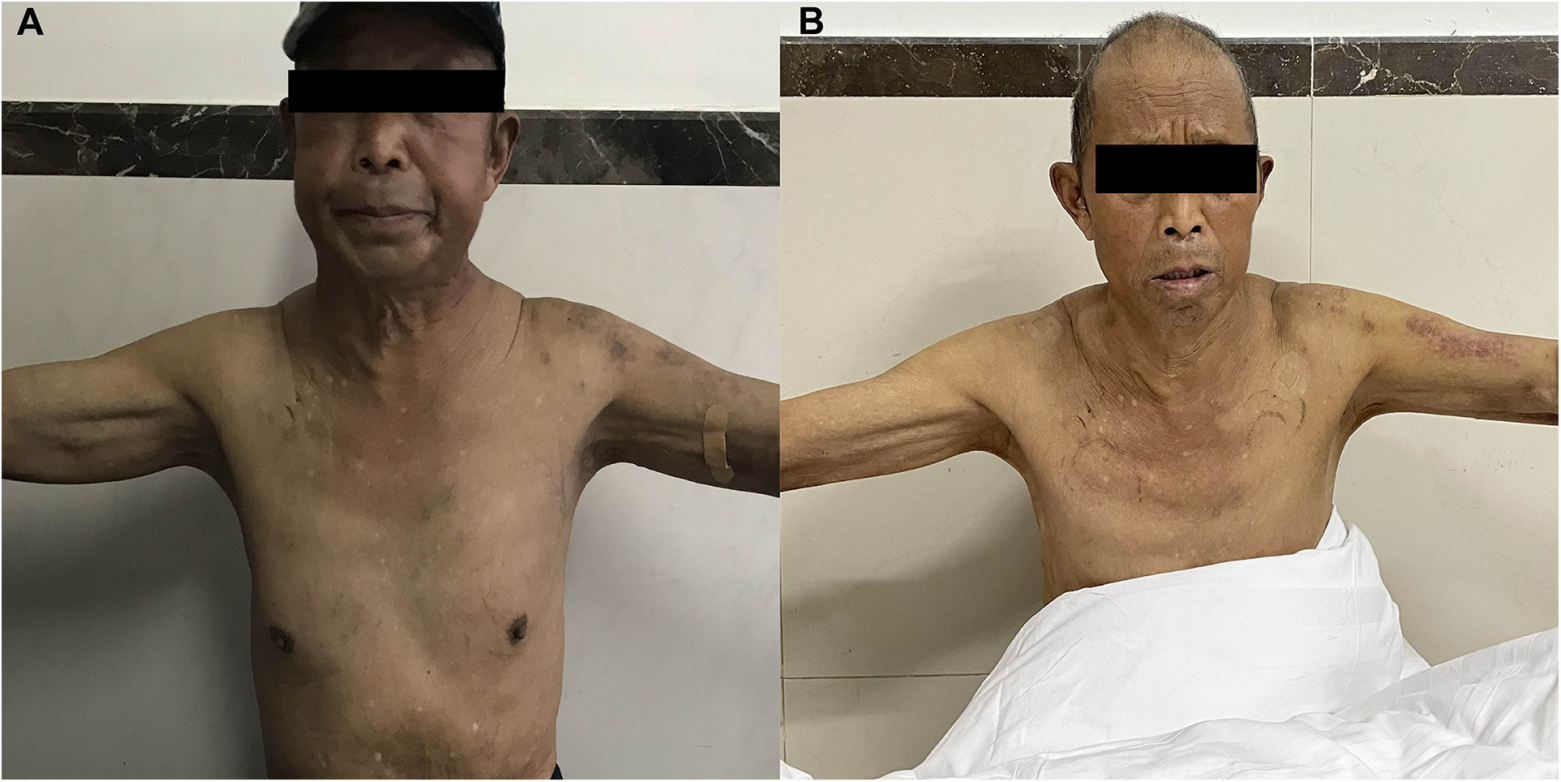
The patient had mild swelling of the face, neck, and upper extremities before procedure **(A)** and these conditions were gradually resolved after revascularization **(B)**.

**Figure 5:**
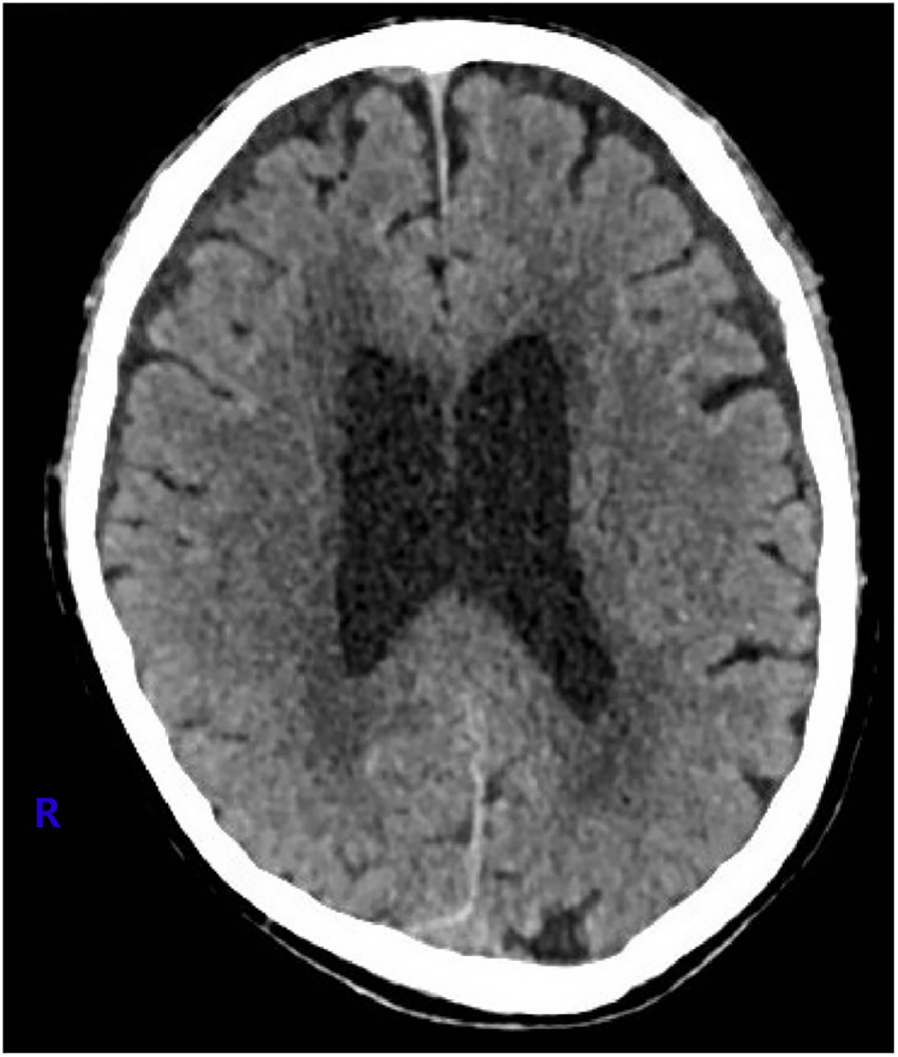
At one year after the procedure, the cranial computed tomography image showed no sign of subdural hematoma.

Given the absence of significant neurological symptoms, conservative management was initiated. For the following three months, preflushing with heparinized saline was performed before each dialysis session, with no additional heparin administered during dialysis. The patient's head and neck swelling gradually resolved (Figure 4), and cranial CT reexamination in April 2021 demonstrated complete resolution of the SDH. No complaint of headache or fatigue from the patient since then. Since June 30, 2021, the patient's dialysis sessions were anticoagulated with nadroparin (2,050 IU). This patient was closely monitored with regular follow-up, no related symptoms were observed. Follow-up imaging every six months revealed no recurrence of SVCS or SDH (Figure 5).

## Discussion

This case highlighted the significant challenges in managing SDH and SVCS in hemodialysis patients. These conditions, although rare, might potentially share underlying pathophysiological mechanisms, such as venous hypertension and hemodynamic fluctuations during dialysis.

SDH represented one of the most life-threatening complications in hemodialysis patients, with 30-day mortality rates reported as high as 35%–39%, depending on the timing and effectiveness of interventions (8). The intracranial bleeding associated with SDH often occurred spontaneously in dialysis patients due to a combination of uremia-induced platelet dysfunction and anticoagulant therapy, both of which impaired normal coagulation pathways (4, 5). Moreover, dialysis-related hemodynamic instability, including rapid fluctuations in blood pressure and fluid shifts, exacerbated the risk of SDH by altering cerebral perfusion and venous pressure (9). In this patient, SDH posed diagnostic and therapeutic challenges. Symptoms can be insidious, such as mild confusion or headaches, which were easily misattributed to uremic encephalopathy or hypertension (10). Timely neuroimaging was crucial to identify SDH before severe neurological deterioration, including coma or brain herniation. The management of SDH in hemodialysis patients required careful consideration of anticoagulation status. Reduction or temporary cessation of anticoagulation during dialysis sessions, while necessary to prevent further bleeding, increased the risk of vascular access thrombosis, creating a delicate balance between competing priorities.

SVCS had become increasingly recognized as a complication of long-term central venous catheter use in hemodialysis patients (3). The thin-walled structure and relatively low pressure of the SVC made it particularly vulnerable to catheter-related injury, which could lead to endothelial damage, thrombosis, and eventual occlusion (11). Obstruction of the SVC impaired venous return from the head, neck, and upper extremities, leading to venous hypertension and the formation of collateral circulation. The clinical manifestation of SVCS varied depending on the speed of obstruction development. Acute obstruction typically resulted in dramatic symptoms such as dyspnea and cyanosis, whereas chronic obstruction, as seen in this patient, presented more with venous distension and persistently mild upper body edema (11). The chronicity of SVCS often complicated its management, as patients may develop extensive collateral circulation that can make interventional procedures more technically challenging.

The resolution of SDH following the successful treatment of SVCS in this patient suggested the potential intercorrelation between these two conditions. The obstruction of the SVC not only influenced venous pressure in the upper body but also increased intracranial venous pressure (7), thereby increasing the risk of SDH. Relieving venous congestion through stenting alleviated SVCS symptoms and reduced intracranial venous pressure, facilitating SDH resolution. Additionally, hemodialysis itself induced significant hemodynamic fluctuations that can exacerbate intracranial pressure changes. Rapid fluid removal during dialysis sessions caused transient hypotension and shifts in blood volume, which could affect cerebral blood flow. In patients with SVCS, where venous drainage was already compromised, these fluctuations may further increase intracranial venous pressure, compounding the risk of SDH (1). Revascularization of the obstructed SVC contributed to reduce these fluctuations and decreased the risk of SDH.

Due to the high surgical risks associated with coagulopathy and uremia, SDH in dialysis patients was often managed conservatively. Blood pressure control, reduction in anticoagulation, and close monitoring were essential to minimize the risk of hematoma expansion (12). In this case, the successful stenting of the left brachiocephalic vein relieved venous congestion, leading to significant improvement in both SVCS and SDH symptoms. The reduction of anticoagulant drugs during the perioperative period may also have contributed to the resolution of SDH. These findings suggested that both addressing venous hypertension and appropriately managing anticoagulation therapy were critical to manage intracranial complications in dialysis patients.

## Conclusion

This case report illustrated the complex relationship between SVCS and SDH in a hemodialysis patient. By addressing the central venous obstruction through stenting, both vascular and neurological symptoms were alleviated, highlighting the potential pathophysiological link between these complications. This case also emphasized the importance of early recognition and management of SVCS to prevent secondary complications such as SDH. Further basic research is needed to elucidate the mechanisms linking these conditions and to develop evidence-based management strategies for affected patients.

## Data Availability

The raw data supporting the conclusions of this article will be made available by the authors, without undue reservation.
